# Joint Analysis of Eye Blinks and Brain Activity to Investigate Attentional Demand during a Visual Search Task

**DOI:** 10.3390/brainsci11050562

**Published:** 2021-04-28

**Authors:** Nicolina Sciaraffa, Gianluca Borghini, Gianluca Di Flumeri, Febo Cincotti, Fabio Babiloni, Pietro Aricò

**Affiliations:** 1 Department of Molecular Medicine, Sapienza University of Rome, Piazzale Aldo Moro 5, 00185 Rome, Italy; gianluca.borghini@uniroma1.it (G.B.); gianluca.diflumeri@uniroma1.it (G.D.F.); fabio.babiloni@uniroma1.it (F.B.); pietro.arico@uniroma1.it (P.A.); 2 BrainSigns srl, Lungotevere Michelangelo 9, 00192 Rome, Italy; 3 IRCCS Fondazione Santa Lucia, Neuroelectrical Imaging and BCI Lab, Via Ardeatina 306, 00179 Rome, Italy; Febo.cincotti@uniroma1.it; 4 Department of Computer, Control, and Management Engineering “Antonio Ruberti”, Sapienza University of Rome, Via Ariosto 25, 00185 Rome, Italy; 5 College of Computer Science and Technology, Hangzhou Dianzi University, Hangzhou 310005, China

**Keywords:** EEG, brain connectivity, attentional demand, eye blinks

## Abstract

In several fields, the need for a joint analysis of brain activity and eye activity to investigate the association between brain mechanisms and manifest behavior has been felt. In this work, two levels of attentional demand, elicited through a conjunction search task, have been modelled in terms of eye blinks, brain activity, and brain network features. Moreover, the association between endogenous neural mechanisms underlying attentional demand and eye blinks, without imposing a time-locked structure to the analysis, has been investigated. The analysis revealed statistically significant spatial and spectral modulations of the recorded brain activity according to the different levels of attentional demand, and a significant reduction in the number of eye blinks when a higher amount of attentional investment was required. Besides, the integration of information coming from high-density electroencephalography (EEG), brain source localization, and connectivity estimation allowed us to merge spectral and causal information between brain areas, characterizing a comprehensive model of neurophysiological processes behind attentional demand. The analysis of the association between eye and brain-related parameters revealed a statistically significant high correlation (R > 0.7) of eye blink rate with anterofrontal brain activity at 8 Hz, centroparietal brain activity at 12 Hz, and a significant moderate correlation with the participation of right Intra Parietal Sulcus in alpha band (R = −0.62). Due to these findings, this work suggests the possibility of using eye blinks measured from one sensor placed on the forehead as an unobtrusive measure correlating with neural mechanisms underpinning attentional demand.

## 1. Introduction

Attention represents a set of cognitive processes that lead to discriminate useful information in a pattern of distractors [1]. It has a decisive role in situational awareness and subsequent decision-making processes: wide pre-attentive processing of environmental features delivers cues for further focused attention because, due to human finite attentional capacities, only a limited amount of information can be processed at a high-level [2]. This is possible thanks to the human ability to move the attentional focus, and this aspect is quite relevant for each multitasking activity performed during everyday life or in most operational working contexts since people can notice only changes inside the focus of attention compatibly with limited cognitive resources [3]. Therefore, both deficits in the intensity aspect of attention (i.e., alertness or vigilance level), due for example to sleepiness, but also issues related to the selective processes, due for instance to distraction caused by irrelevant sources of information, could have negative effects on decision making.

In this context, it is important to gain a better understanding of attentional processes to make them measurable, even online, and to predict attentive deficits during everyday activities.

The level of attentional demand (or the amount of attentional investment) could be assessed by means of questionnaires or by evaluating the subject’s performance. In both these cases, the measures are made ex-post and cannot be used to predict attentional defects. The neurophysiological evaluation of attention, mostly employing eye and brain activity measurement, can allow continuous monitoring of the attention level, acting both as feedback for the user and to eventually adapt the features of the system he is interacting with [4,5]. Electroencephalography (EEG), functional near-infrared, functional magnetic resonance imaging (fMRI), and magnetoencephalography are the techniques typically used for recording brain activity. On the one hand, techniques like fMRI require room-sized equipment that prevents them from being usable in everyday activities [6]; however, fMRI allows investigation of cortical and subcortical activities. On the other hand, the EEG, thanks to its portability and high time resolution, aids in looking at brain activity in different oscillatory bands [7]. Moreover, the high-density EEG allows to enhance the EEG spatial resolution and, thanks to the employment of source reconstruction methods, to have access to cortical and subcortical structures [8]. Alongside this, eye activity is usually recorded by means of eye-tracking or electrooculography (EOG). From the instrumental point of view, these techniques are much less obtrusive than high-density EEG; however, they cannot give direct access to the neurophysiological process.

In this framework, a joint analysis of brain activity and eye blinks provides an ideal neuroscientific model to investigate the association between brain mechanisms and behavior [9]. Therefore, this study aimed to jointly analyze brain and eye blinks during a task eliciting different levels of attentional demand. In the literature, the need for a joint analysis of brain and eye activity has been felt in several fields. For a multimedia analysis, a joint analysis would allow merging human perception and brain reactivity, which are considered both important information sources [10]. In the clinical field, providing new information on how the structural, functional and behavioral modifications in early attention abilities reported in Autism Spectrum Disorder patients are connected is crucial [11]. In different applied contexts, eye movements are assumed to work as distinct points in information processing, necessary to segment data and perform a time-aligned analysis of brain activity [12]. In fact, eye and brain activities have been already analyzed jointly in a time-locked configuration and eye blink-related potentials have provided reliable information on cognitive processing due to the interrelation between eye blinking and the dopaminergic system. For example: (i) microsaccade-locked event-related potentials have been used to assess mental workload [13]; (ii) fixation duration, pupil size, and event-related potentials locked to the onset of fixation or saccade have been used to classify two levels of cognitive task showing complementary contributions of eye and brain activity measures [14]. Moreover, the effects of word predictability on eye movements and fixation-related brain potentials has been demonstrated [15]. Finally, the development of neural mechanisms of attention shifts during infancy and covert/overt attention in adults was investigated by combining eye-tracking and event-related potential analysis [16,17].

In our study, eye blinks and brain activity have been analyzed jointly without imposing a strict timing task, the need for which has already been expressed in [18]. This approach is particularly valuable to show how brain dynamics underlying perceptual and cognitive processes over time. In this regard, the number of works is as far as lower than the aforementioned time-aligned based analysis. One of the first works has shown an inverse correlation between eye blink rate and EEG alpha power after sleep deprivation [19]. More recently a correlation between saccade amplitude, saccade velocity, and blink rate with EEG power in delta, theta and alpha band during a vigilance task has been analyzed [20]. It was found that saccade measures, frontal midline theta and frontal theta to parietal alpha ratio correlate positively with vigilance level, while blink rate and relative delta power correlate negatively with vigilance level. Liu et al. analyzed time series of eye movement and EEG channel activity acquired while subjects were watching a movie trailer. They found that eye and brain activity are more correlated when a higher quality movie trailer is watched [10]. In the clinical field, an opposite association between theta-beta ratio with look duration for typically developing children and children affected by Autism Spectrum Disorder has been found [21]. Finally, not only EEG power but also the coherence between brain activities has been correlated with eye-tracking measures (fixation duration) to investigate the neurophysiological underpinnings of gaze processing [11].

In order to achieve our goal, attentional demand was manipulated using a Conjunction Search Task (CST), a stimulus set paradigm based on the recognition of a target object in a pattern of similar distractor objects with a low influence on memory load and general cognitive functions [22]. The target differentiates from others for one or more features. If the object can be recognized with one feature (i.e., shape or color) the recognition happens in a *pre-attentive state*, because the object has a “pop-out” effect and it does not need, or at least involves only a few, attentional resources [23]; its presence could also be detected covertly (i.e., without gazing at the stimulus itself). The necessity to recognize the “conjunction” of more than one feature implies that an *attentive state* will be used to recognize the object and a serial searching (overt attention) of the objects, and more attentional resources are usually required [24]. Higher attentional demand has been associated with conjunction search due to top-down voluntary allocation of attention to features, opposite to “pop-out effects” [25]. According to the Feature Integration Theory [26], the *conjunction search* corresponds to the high attentional demand level because noticing the “conjunction” of more than one feature would demand more attentional investment than that required during the *one feature search*. This hypothesis has also been experimentally confirmed by [27] and it has been used to analyze two long-term attentional states in [28]. Thus, CST allows a very different behavioral response at the two levels using comparable visual information. 

From the perspective of ocular activity, task demand modulates the numbers and duration of eye blinks, saccadic movements, and gaze fixation [29]. Among these, we focus herein on eye blinks because they are easy to measure without additional instrumentation and their relationship with task demand has been deeply demonstrated. Several conflicting results have shown that there is not an unambiguous effect of task demand and stimulation on eye blink frequency and duration. A decrease in blink rate has been associated with increased information processing [30], whereas an increase in blink rate has been associated with both a low [29] and high [31] cognitive demanding task. In fact, the latter depends on the nature of the task itself [31]. For example, during an auditory task the blink rate increases as the difficulty increases [32]; eye-blink rate decreases due to the increase of difficulty during a mental arithmetic task but not during a letter-search task [33]. Similar unconverging results have been found for blink duration: a higher blink duration has been associated with less demanding tasks [29] and with a higher visual load [34], whereas decreasing blink duration has been associated with greater visual information processing [34]. Therefore, we hypothesized that because low and high levels have been characterized by a comparable visual load, both eye blink rate and duration should vary according to the task demand during a visual stimulation; that is, they should decrease during the high demanding condition.

Largely due to fMRI-based experimental protocols, it is already known that the different behaviors associated with the two CST levels reflect specific neural correlates in terms of both brain activity and connectivity [35]. The top-down voluntary allocation of attention is guided by the dorsal attention network (DAN) centered in the frontal eye fields (FEF) and intraparietal sulcus (IPS). IPS and the FEF exert influence over the visual area, and more in general over sensory areas of the brain, during spatial orienting [36] and top-down processes [37]. When relevant stimuli occur unexpectedly or they appear outside the cued focus of spatial attention, the attention-related processes are guided by a ventral attention network (VAN) consisting of temporoparietal junction (TPJ) and ventral frontal cortex [36,38]. The TPJ has been considered to be like a filter, allowing goal-driven behavior [36] and it plays a hub role when eye movements and attention are disassociated or when attention is not directed towards the focus [35]. From the perspective of brain activity, the conjunction condition implies an overall increase in IPS and FEF activation and a decrease in the ventral brain areas and TPJ activation [25,39]. EEG-based evidence found that tasks requiring enhanced top-down processing are coupled with increased beta activity and decreased alpha activity [40]. Moreover, several works have found that increased activity of DAN in the theta band is associated with increased task demand [41,42]. In the current work, high-density EEG recordings enabled the application of a standardized low-resolution brain electromagnetic tomography (sLORETA) [43] method for reconstructing the brain activity in the aforementioned regions of interest (ROIs). In parallel, the analysis of brain activity at the scalp level has been performed. Due to the similarity of our experimental design with that described in [28], their results obtained at the scalp level are noteworthy. They found a significant increase in brain activity over the frontal, central and parietal regions in the theta band during the high demand condition compared to the low, whereas they did not find a modulation of brain activity in the alpha and beta band even if it had been hypothesized.

Due to these findings, we hypothesized that during the high attentional demand condition, brain activity should increase in the theta and beta band over the frontal and central brain areas, and decrease in the alpha band in frontal, central, and parietal areas. Moreover, DAN activation should be higher and TPJ activation should be lower during high, compared to low, conditions.

Finally, to characterize the brain network associated with low and high attentional demand, brain connectivity between ROIs has been estimated by partial directed coherence (PDC) [44] and graph theory indices have been computed. Previous studies showed that the attentional demand modulates brain connectivity between the frontal-parietal network and visual areas in theta, alpha, and beta bands [41,45]. In particular, high demand induces increased interregional connectivity in the DAN [35,41], increased influences of the DAN on visual areas [37], and strengthened VAN-DAN connectivity [36]. Therefore we hypothesized that the high attentional demand condition should be characterized by increased connectivity between dorsal, ventral, and visual areas.

Therefore, this work (i) analyzed the effects of attentional demand on eye blinks, brain activity, and brain network features starting from high-resolution EEG signals, and (ii) tested the association between endogenous neural mechanisms underlying attentional demand and eye blinks, which means eye behavior is correlated to brain activation and defined brain network features, without imposing a time-locked structure to the analysis.

## 2. Materials and Methods

### 2.1. Participants

The study involved twelve healthy subjects (27 ± 3 years old), 6 males and 6 females, recruited on a voluntary basis. The experiments were conducted following the principles outlined in Declaration of Helsinki of 1975, as revised in 2000. It has been approved by the Ethical Committee of Fondazione Santa Lucia. Informed consent was obtained from each subject on paper, after the study explanation.

### 2.2. Conjunction Search Task

**Figure 1:**
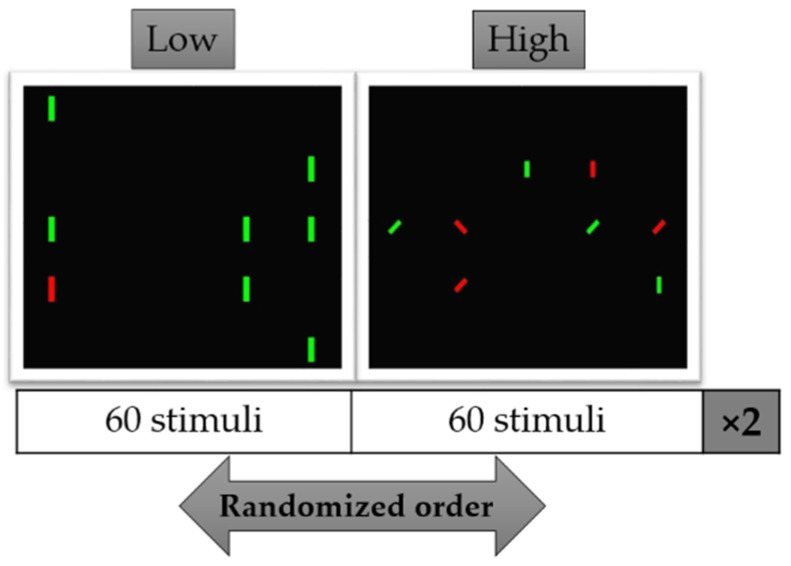
Conjunction Search Task. In both Low and High levels the target is a red vertical bar. In the Low condition, the distractors were green vertical rectangular bars. In the High condition, the distractors were vertical and 45° rotated green bars and 45° rotated red rectangular bars.

The Conjunction Search Task (CST) was divided into two blocks comprehending 120 trials each, in turn divided into two conditions. In particular, in each block participants performed 60 trials of two different conditions requiring different levels of attentional demand. One of them was a pre-attentive level based on a ‘target’ search task by considering one feature (color). This condition has been defined as a Low condition according to the lower attentional demand required. In contrast, the High condition consisted in the conjunction search based on two features (color and orientation) that require a higher attentional demand. The sequence of the two conditions (Figure 1) was randomized for each participant to avoid habituation and expectation effects. The participants performed 10 practice trials per condition before starting with the experiment. When the practice was done, and the participant was ready to start, the experimental trials began.

The 60 trials of each condition were randomized in 30 targets and 30 non-targets. For each trial, the goal was to find, if present (target trial), the vertical red bar (target) among distractors and to react, as fast as possible, by pressing the space bar. No action was required when the target was not presented (non-target trial). All the stimuli were presented against a black background on a 25 position matrix filled with 8 elements: 7 distractors and 1 target (target trial) or 8 distractors (non-target trial). The matrix was presented to the participants for 2 s and between two trials a fixation cross was presented at the center of the screen for a random interval between 0.25–1 s. Both target and distractors were rectangular bars (size: 0.5 × 1.6 visual angle) and the target was always a vertical red bar. In the Low condition, the distractors were green vertical rectangular bars. In the High condition, the target was defined by two different features, color and orientation, the distractors were vertical and 45° rotated green and red rectangular bars. The task has been implemented in Mathworks MATLAB using Psychtoolbox software.

### 2.3. Behavioural Measures

To assess both accuracy and reaction time of the user through one synthetic index, we used the Inverse Efficiency Score (IES [46]) defined as reaction time (ms) for corrected responses divided by the percentage of correct responses. IES has been used to compare the performance across different levels of attentional demand required during CST (Low and High). According to its definition, lower values of IES are expected for the low demanding level because answers are supposed to be faster and more accurate than at the High level.

### 2.4. Brain Activity Measurement

The EEG signal was recorded by 61 Ag-Ag/Cl passive electrodes by means of a digital monitoring system (BrainAmp, Brain Products GmbH) with a sampling frequency of 250 Hz. All the electrodes were referred to both earlobes and their impedances were kept below 10 kΩ. The EEG signals were firstly band-pass filtered with a fifth-order Butterworth filter between 1 and 30 Hz and then segmented into epochs of 1 s. This specific epoch time-length was selected to have a condition of stationarity of the EEG signal [47]. Each EEG epoch and each channel has been analyzed for the detection of general artifacts (i.e., muscular, instrumental). Ocular artifacts have been detected through the Reblinca algorithm and removed [48]. According to [49], each epoch with an amplitude higher than ±80 μV or a slope trend higher than 10 μV/s was considered an artifact and was replaced by NaN values. For each epoch, the Power Spectral Density (PSD) was calculated. Then, the EEG frequency bands were defined considering the Individual Alpha Frequency (IAF) estimated for each subject [50]. The IAF corresponds to the peak in the alpha band (typical IAF value is 10 Hz) obtained from the power spectrum of individual EEG signal over parietal sites during a rest condition. Before performing the experimental tasks, the subject was asked to keep his/her eyes closed for one minute, because the alpha synchronization, and thus the spectrum peak, is maximum during this condition. Therefore, theta (IAF − 6 ÷ IAF−2), alpha (IAF − 2 ÷ IAF + 2), and beta (IAF + 2 ÷ IAF + 16) bands have been defined accordingly. 

To assess the spatio-spectral differences of brain activity between the Low and High condition, the PSD values have been averaged in the Theta, Alpha, and Beta band. Therefore, a Wilcoxon signed-rank test has been performed for each channel and each band on the PSD values of the subjects. The results have been corrected for multiple comparisons through the False Discovery Rate (FDR) method [51].

### 2.5. Brain Connectivity Estimation

**Figure 2:**
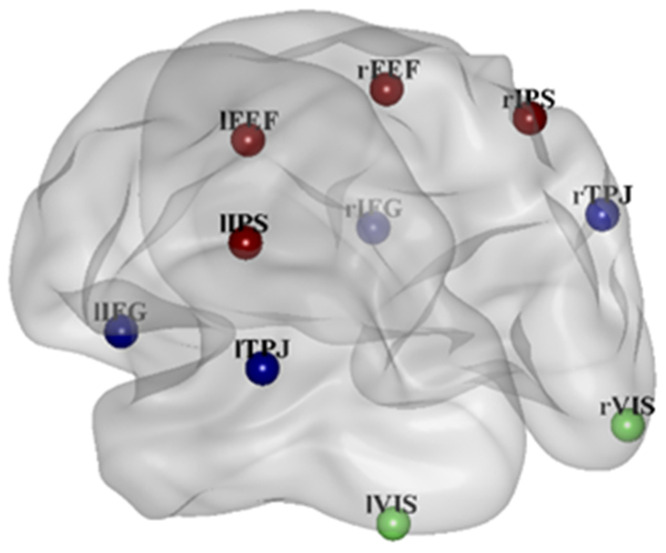
3-D representation of ROIs. Ventral attention network is in blue (Inferior Frontal Gyrus (IFG), Temporal Parietal Junction (TPJ)). Dorsal Attention network is in red (Frontal Eye Field (FEF), Intraparietal sulcus (IPS)). The visual areas are in green.

The artifacts-free EEG signals have been used to reconstruct the brain source activity by means of the sLORETA method [43]. This method has been chosen because it is characterized by high accuracy in the localization of brain sources; in fact, the global average of localization errors remains below the unit. The MNI-152 realistic head model has been used [52]. The individual EEG data have been provided to the software with the electrode locations file to match them with the model [53]. Once the reconstruction of the activity for the whole brain was computed, sLORETA was used to estimate the brain activity in the single nearest voxel of 10 selected ROIs. These ROIs have been selected from the literature review of works analyzing the conjunction search task through fMRI [35] and the role of the dorsal and ventral attention network [36]: Inferior Frontal Gyrus (IFG), and Temporal Parietal Junction (TPJ), representing the Ventral Attention Network; Frontal Eye Field (FEF), and Intraparietal sulcus (IPS), representing the Dorsal Attention Network; and visual areas (VIS) are the brain areas mainly representative of the attentional networks (Table 1 and Figure 2 for graphical representation). The estimated cortical activity in the bands of interest have been averaged in each ROI for both Low and High conditions and the difference has been compared through a Wilcoxon signed-rank test corrected for multiple comparisons by the FDR method [54].

Brain connectivity analysis provides the information flows exchanged between brain areas. It has been estimated through Partial Directed Coherence (PDC) [44]. The PDC is a multivariate spectral measure used to determine the directed influences among any given pairs of signals obtained; in this case, from the estimation of brain activity in the 10 selected ROIs. This estimator reflects a frequency version of the concept of Granger causality [55]. The reconstructed source activity, in the linear signal processing framework, can be described as a multivariate process of *M* processes $Y\left(n\right)={\left[y_{1}\left(n\right),\ldots ,y_{M}\left(n\right)\right]}^{T}$. Assuming that the following multivariate autoregressive (MVAR) process is an adequate description of *Y*:

(1) $$Y\left(n\right)=\overset{p}{\underset{k=1}{\sum }}A\left(k\right)Y\left(n-k\right)+E\left(n\right),$$

where:

- $A\left(k\right)$ are the *M* × *M* coefficient matrices in which the element $a_{ij}\left(k\right)$ describes the dependence of $y_{i}\left(n\right)$ on $y_{j}\left(n-k\right)$ (i,j=1,…,M;k=1,…,p). In this case, the present value of a specific process can be described as a linear function of the *p* past values of all processes, that is the model order, which in this work was estimated through Akaike Information Criterion (AIC) [56];
- $E\left(n\right)={\left[u_{1}\left(n\right),\ldots ,u_{M}\left(n\right)\right]}^{T}$ is a vector of *M* zero-mean input processes. It is assumed to be composed of white and uncorrelated noises, which means that the correlation matrix of ***E***(*n*) is equal to the covariance matrix for *k* = 0 and it is zero for each lag *k* > 0. Under the assumption of strict causality (e.g., the absence of instantaneous effects), the input white noises are uncorrelated, even at lag zero and their covariance matrix reduces to the diagonal matrix $\Sigma =\mathrm{diag}\left(\sigma_{i}^{2}\right)$.

In this scenario, the spectral representation of an MVAR process can be obtained taking the Fourier Transform (FT) of the representation which yields to $Y\left(f\right)=A\left(f\right)Y\left(f\right)+E\left(f\right)$ where $Y\left(f\right)$ and $E\left(f\right)$ are the FTs of $Y\left(n\right)$ and $E\left(n\right)$, and the *M* × *M* transfer matrix and coefficient matrix are defined in the frequency domain as $A\left(f\right)=\sum_{k=1}^{p}A\left(k\right)e^{-j2\pi fkT}$ where T represents the sampling period of the discrete time process. By exploiting [57] and by following the methodology introduced in [44], PDC can be defined as:

(2) $$\pi_{ij}\left(f\right)=\frac{{\bar{A}}_{ij}\left(f\right)}{\sqrt{\sum_{m=1}^{M}{\left|A_{mj}\left(f\right)\right|}^{2}}}$$

This quantity represents a measure of the direct influence of $y_{j}$ onto $y_{i}$ and is a complex value. Due to this, the squared modulus of PDC is used in this study to measure the connectivity in the frequency domain. The squared measure takes values between 0 and 1, representing absence and full connectivity respectively. Besides the estimation of the strength of connectivity between a couple of processes, it is necessary to assess their statistical significance to establish the existence of a direct link from the *i*-th process to the *j*-th process [58]. The significance of PDC was tested using a theoretical distribution of PDC derived from the Asymptotic Statistics theory, which assumes that, in the presence of connectivity, PDC distribution for an infinite number of data samples tends asymptotically towards a Gaussian distribution [59]. Then, the 95th percentile of each distribution, representing the threshold, extracted for each link and frequency bin, was compared with each estimated PDC value in order to assess the statistical significance.

Therefore, in this work, firstly the weak stationarity of the EEG time series was tested by using the approach proposed in [60]. Secondly, brain connectivity patterns were estimated for each experimental condition (Low and High). Thirdly, the PDC values filtered through Asymptotic Statistics were averaged within the three considered EEG frequency bands.

The estimated connectivity pattern can be represented by a graph where each ROI is a node and the connections are the edges depicting the interactions among the nodes’ activity. Each edge is characterized by a weight and a direction. The role of each node in a network can be analyzed by means of an index derived from graph theory. In particular, the importance of a node in a network could be easily identified with its degree, that is, the number of connections involving that node: the higher the number of edges, the higher its impact on the other nodes serving as a “hub” in a network will be [61]. In this work, the role of each node has been differentiated according to its belonging to a sub-network (i.e., the dorsal anterior network, the ventral attention network, and the visual areas) computing the participation index. The participation index (PI) is a measure of the diversity of inter-modular connections of individual nodes. The PI of a node is low if most of that node′s connections are within a single community, while it is high for nodes that serve as connectors between different modules [62]. The PI has been computed considering separately the connections incoming (participation in) and the connections outcoming (participation out) for each node. Moreover, for each node the strength of the connections involving (and therefore incoming and outcoming) the node itself has been computed. The PI and the strength during the different conditions have been compared through a Wilcoxon signed-rank test and corrected for multiple comparisons by the FDR method [54].

### 2.6. Eye Blink Measurement

Eye activity has been analyzed in terms of blink-related features. The blink detection has been performed using a variant of the BLINKER pipeline [63]. In its original implementation, the BLINKER algorithm selects the best channel among an arbitrary number of EEG channels, allowing the optimal identification of blinks. However, we forced the BLINKER algorithm to use an Fpz channel to detect blinks. We selected the Fpz channel, instead of a bipolar channel, for EOG activity, since (i) this EEG channel has been already demonstrated to be a reliable regressor for eye-blink activity; and (ii) in order to simulate a very low invasive system of acquisition using one of the EEG electrodes, in particular on the forehead, and to record brain and eye activity together [48]. According to the BLINKER pipeline, the Fpz signal has been bandpass-filtered between 1 and 20 Hz. Potential blinks have an amplitude 1.5 times signal standard deviation, duration higher than 100 ms and interblink interval higher than 50 ms. Among the potential blinks, only those showing a correlation with the tent-like shape, representing the ideal blink shape, higher than 0.9 have been considered. The last check selects the blinks with a Positive Amplitude Ratio higher than 3 to remove saccades. In fact, due to the visual component of the task, the saccades could represent a task-related confound and do not provide any information strongly related to attention. For each condition, the number of blinks per minute (Eye Blink Rate-EBR), and the blink duration during one-minute-long windows have been computed. Each of the eye-related parameters has been computed on a window length of one minute, in order to obtain the stability of the measure, as highlighted in [64]. A Wilcoxon signed-rank test has been performed to assess the statistical differences between Low and High conditions.

### 2.7. Correlation between Eyes and Brain Features

The eye activity features have been correlated with the features related to brain activity (PSD in the bands of interest) and with those related to brain connectivity (PI).

In the first case, the correlation between spatio-spectral brain features and eye-related parameters of each subject has been performed binwise, computing the Spearman’s rank correlation coefficient. Due to this approach, an increasing type I statistical error is expected. To face this effect, the Descriptive Data Analysis procedure, based on the definition of Ruger’s area for neurophysiological data, has been applied [65]. In the *number of channels x frequency bins* domain is defined as Ruger’s area each continuous region showing *p* < 0.05. To correct for multiple comparisons, these regions are further tested: to refuse a global null hypothesis, it is necessary that half Ruger’s area has *p* < 0.025 and that one-third of the Ruger’s area has *p* < 0.0167. We did not consider Ruger’s area with fewer than 2 bins.

Secondly, to explore the existence of a relationship between the brain network properties and the eye blinks features, the repeated measures correlation has been computed between the average values of the eye blinks parameters and the participation index. Unlike simple correlation, repeated measures correlation does not violate the assumption of independence of observations, and has a greater statistical power in estimating the association shared among individuals [66].

## 3. Results

### 3.1. Behavioural Measures

**Figure 3:**
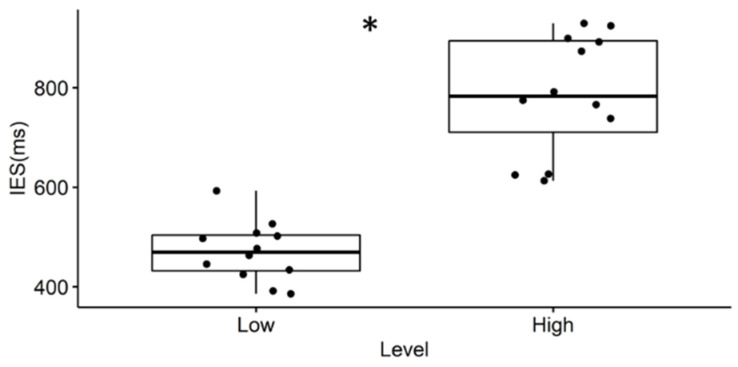
Results of Behavioral Measures. The higher the Inverse Efficiency Score (IES), the lower the performance of the subjects when a greater attentional demand is required. The asterisk (*) means *p* < 0.05.

The Wilcoxon signed-rank test performed between the two conditions provides a significant result (Z = −3.094, *p* = 0.0022): IES during High level is significantly higher compared to the Low level (Figure 3).

### 3.2. Neurophysiological Correlates

Table 2 shows the results for eye blinks. The increase in attentional demand is associated with a significant decrease in the number of blinks per minute (*p* = 0.004) and there is not a significant difference in blink duration (*p* = 0.386).

**Figure 4:**
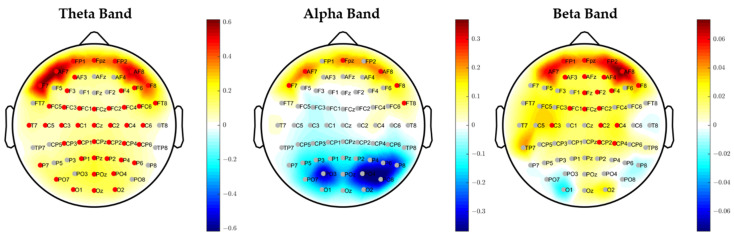
Average over the population of the difference between brain activation in the high condition compared to the low condition in the Theta, Alpha, and Beta band. The color bar represents the difference in power spectral density value (negative values mean that the brain activity in such area is on average higher in low condition compared to high). The electrodes significantly (*p* < 0.05 FDR corrected) more active at the High level with respect to the Low level are marked in a red color, those more active at the Low level with respect to the High level are marked in a blue color (none), and the insignificant level is marked in grey.

Figure 4 shows the average over the population of the difference between brain activation in the high condition compared to the low condition in the three bands. The color bar represents the difference of PSD values (negative values mean that the brain activity in such area is on average higher in low condition compared to the high condition). According to the statistical analysis, the electrodes significantly (*p* < 0.05 FDR corrected) more active at the High attention level with respect to the Low attention level were marked in a red color, those more active in the High attention level compared to Low were marked in a blue color, and the insignificant level was marked in grey. The results show that there is a general higher activation of brain activity in all three bands when a higher level of attention is required, except for the parietal and occipital areas in the alpha band. Such an increase in the theta band is diffuse on the whole scalp, and it is localized in the anterofrontal area in alpha band, and on the frontal and centro-parietal areas in the beta band. 

**Figure 5:**
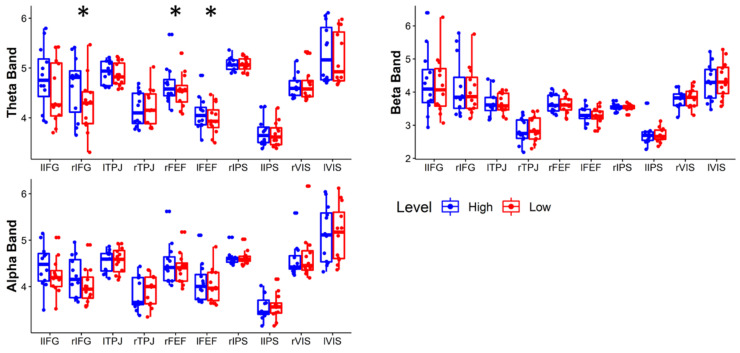
Wilcoxon test between the ROI activation in the Low and High conditions in Theta, Alpha, and Beta bands. The asterisk (*) means *p* < 0.01252 (FDR corrected).

By applying sLORETA methods, it has been possible to estimate cortical activation. Figure 5 shows the statistical comparison of the ROIs activity in High and Low conditions. In the theta band, the rIFG (*p* = 0.0068), lFEF (*p =* 0.0068), and rFEF (*p* = 0.0068) show significantly higher activation in the High condition compared to the Low. 

**Figure 6:**
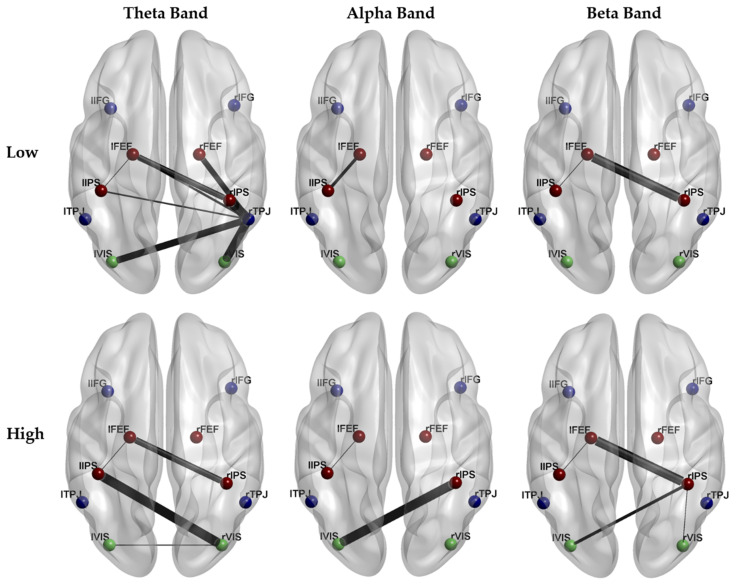
Average over the population of Brain Connectivity Networks in Theta, Alpha, and Beta bands in case of Low and High attentional demand. The thickness of each connection is representative of its weight averaged over the population.

**Figure 7:**
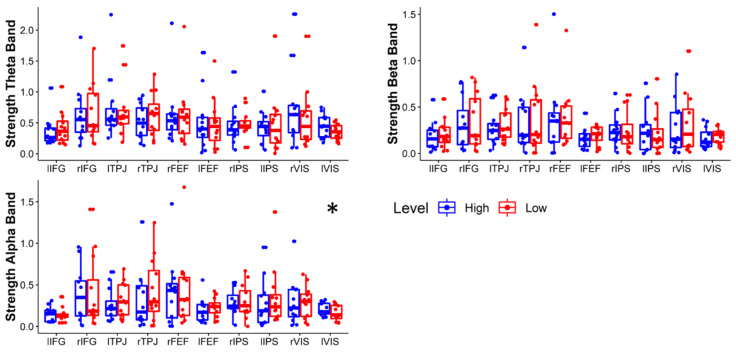
Wilcoxon test of the strength in Theta, Alpha, and Beta bands between Low and High attentional demand. The single asterisk (*) means *p* < 0.01252 (FDR corrected).

**Figure 8:**
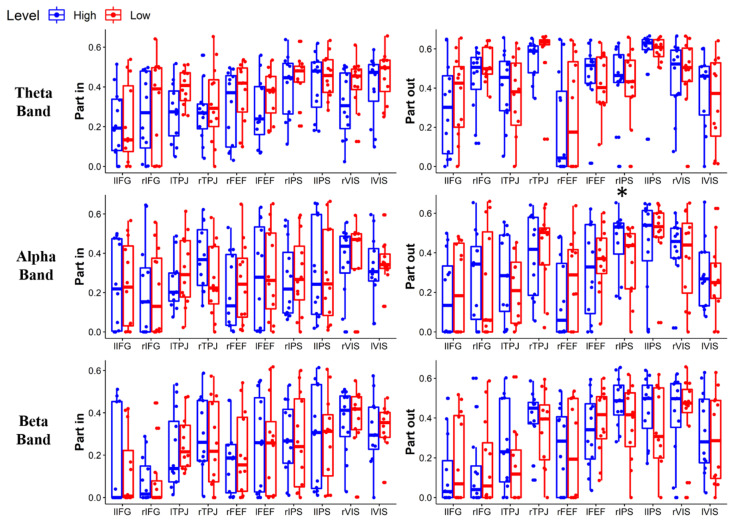
Wilcoxon test between Low and High attentional demand of the participation in (**Left**) and the participation out (**Right**) indexes for each ROI and band (**rows**). The single asterisk (*) means *p* < 0.01064 (FDR corrected).

Figure 6 shows the brain connectivity networks characterizing the High and Low conditions in theta, alpha, and beta bands. Each connection represents the average value of the connection between two ROIs over the population. The role of each ROI in the network has been quantitatively characterized by graph theory indices, in particular by the strength and participation index. Figure 7 shows the statistical comparison of strength values for each ROI in the High and Low condition. Only the strength of lVIS in the alpha band significantly increases in the High condition (*p* = 0.012). Figure 8 shows the statistical comparison of PI indexes between Low and High conditions. Considering the correction for multiple comparisons, the participation out of rIPS in the alpha band (Z = 74, *p* = 0.006) is the only value providing *p* < 0.01064. Other marginal significant values in the theta band include a significant increase in participation in rFEF (Z = 10, *p* = 0.022) and an increase in participation out of rTPJ (Z = 13, *p* = 0.041) related to a decreasing attentional demand. The rIPS shows a significant reduction of participation out, also in the beta band (Z = 64, *p* = 0.049).

### 3.3. Correlation between Measures

**Figure 9:**
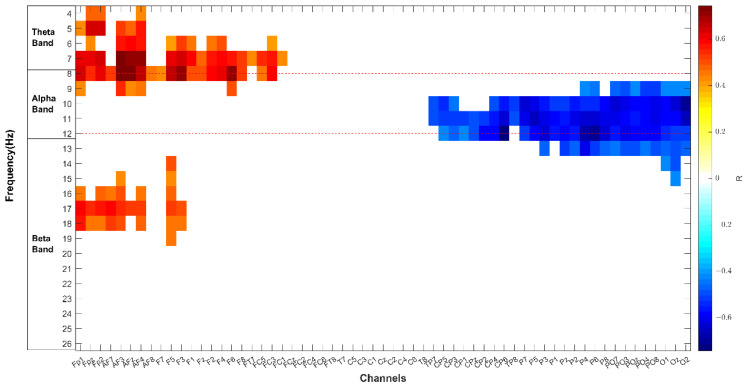
Results of correlation analysis according to the Descriptive Data Analysis procedure. Results of corrected correlation between EEG power and EBR has been represented in channels-frequency domain. The color bar represents the value of Spearman correlation (R). The horizontal red dashed lines highlight frequency where maximum and minimum values of correlation have been found (respectively 8 Hz and 12 Hz).

To test the association between the features related to eye-blink and to brain, different measures of correlations have been computed. Figure 9 shows correlation results between EEG, PSD, and EBR according to Rüger areas correction. A first significant Rüger area shows a positive correlation of EBR with theta power and low alpha power over frontal electrodes (maximum correlation *R* = 0.7426 at 8 Hz on AFz).

In the beta band, two significant Rüger areas show a positive correlation (from 15 to 18 Hz) over frontopolar and anterofrontal electrodes and from 14 to 19 over left frontal electrodes. 

The last significant Rüger area shows a positive correlation of EBR with Alpha power and low Beta power over centroparietal and parietal electrodes (minimum correlation *R* = −0.7348 at 12 Hz on CP6).

**Figure 10:**
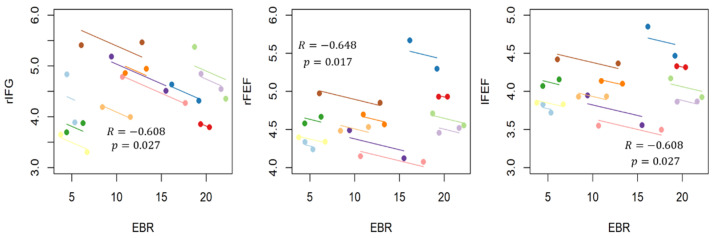
Results of repeated measures correlation between blink parameter Eye Blink Rate (EBR) and the activity of those ROIS which have shown significant modulation of their activity, i.e., rIFG, lFEF, and rFEF in the theta band.

The ROIs provided significant modulation of their activity and have been correlated with EBR, performing a repeated measures correlation (Figure 10). Significant negative moderate correlations have been found in all three cases. 

**Figure 11:**
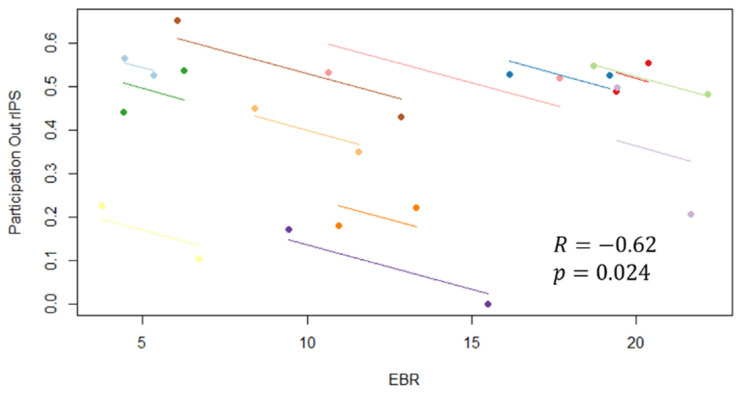
Results of repeated measures correlation between blink parameter Eye Blink Rate (EBR) and participation out of rIPS in the alpha band.

The ROIs provided significant modulation of their strength (i.e., lVIS in the alpha band) and participation index (i.e., participation out in alpha band of rIPS) have been correlated with the significant eye blinks related feature (EBR). Figure 11 shows the plot of repeated measures correlation: a significant negative moderate correlation (*R* = −0.62, *p* = 0.02) of the participation out of rIPS in the alpha band with EBR has been found. The strength did not provide a significant correlation (*R* = −0.324, *p* = 0.28).

## 4. Discussion

The rationale of the present work was to analyze attentional demand in terms of brain connectivity, brain activity, and eye blinks, and to investigate the association among these different measures. Attentional demand has been manipulated using the Conjunction Search Task. This task allowed us to obtain a Low and High attentional demand, associated respectively to the “pop-out” effect and to the “top-down control”.

The results seem to attest that this experimental hypothesis is true both from a behavioral and a neurophysiological perspective. In fact, participants have reacted faster and more accurate during the Low condition, when the single (color) feature has a pop-out effect, with respect to the High condition, when two (color/ orientation) features need to be recognized (Figure 3). We found that in the high attentional demand condition compared to the low condition (i), the eye blink rate significantly decreased (Table 2); (ii) the brain activity significantly increased over the scalp in the theta band, localized on the anterofrontal areas in the alpha band and on the frontal and centro-parietal areas in the beta band (Figure 4); (iii) rIFG, rFEF, lFEF were significantly more activated (Figure 5); (iv) connections involving lVIS were significantly stronger (Figure 7); (v) the participation out of rIPS in alpha band is higher. Contrary to what has been hypothesized, we did not find a significant modulation of eye blink duration and a significant desynchronization of brain activity in the alpha band. We further found that eye blink rate, brain activity and connectivity features do not only show a significant modulation according to the attentional demand but also, they correlate.

With respect to the previous studies, in the current work both eye and brain activity have been analyzed without imposing a time-locked structure on the analysis, a necessity emerged in several different fields, as described in [18]. Therefore, this work fits into a methodological framework, whose number of works in the literature are lower than those using a time-locked analysis and more research is needed to investigate cognitive processes during long-term stimulations [67].

Regarding the findings related to eye blink activity, it is noteworthy to highlight that one EEG channel placed on the forehead (Fpz electrode, referenced to earlobes) has been chosen to measure eye blinks in order to simulate a very low invasive detection system (i.e., one bipolar channel). It has been already proved that the Fpz electrode provides a reliable regressor of ocular activity [48] and allows us to monitor the number and the duration of blinks [63]. Even if the use of blink parameters is affected by strong variability both within and between subjects, in the current work a decrease of eye blink rate in the high condition has been observed, coherent with the literature, which associates this decrement with an increase in information processing [30,34]. However, the cognitive demand is not the only intrinsic factor affecting eye blink parameters: differences in visual load can induce an unexpected variation in both eye blink rate and duration [68]. Therefore, because for our experimental design we assumed (according to [28]) a comparable visual load between high and low conditions, we hypothesized that there was not enough of a difference between the conditions to induce a significant modulation in eye blink duration. Indeed, no difference in eye blink parameters was found between low and high conditions, even in [28].

The spatio-spectral distribution of brain activity observed in the high attentional demand condition in the current study is the one that is typically associated with a high cognitive load, namely an increase in activity in frontal areas [31]. On the scalp, this increment is localized on frontal and centroparietal areas in the beta band, mainly in the right hemisphere coherently with the known about its active role in optimal selection [69,70]. Also the increment of brain activity in the theta band in parietal areas has been already associated with attentional demand [71]. In contrast, we did not find a significant desynchronization of parietal alpha associated with high cognitive demand, even if there is a nonsignificant reduction of that activity, whereas we found a significant increase of anterofrontal activity in the alpha band, which could reflect the enhanced attentional control mechanisms typical of the conjunction search condition [72].

The significant brain activity observed in frontal regions in the theta band reflects the significant activation of rIFG, rFEF, lFEF in high attentional demand conditions compared to the low condition. On the one hand, this finding reflects the increase in the dorsal attention network activation during more difficult tasks and the top-down control (characterizing the High condition [41]). On the other hand, the increase in rIFG activity belonging to the ventral attention network could be considered an unexpected result because the IFG was shown to be deactivated during top-down control [36]. The activation observed is probably due to the long-term approach of this analysis that does not allow us to differentiate between the attentional mechanisms, but rather the different amount of attentional demand. Whereas the time-locked studies (based mainly on Event Related Potentials) have already shown that serial shifting of attention strains attentional control, only recently has it been demonstrated that when subjects are engaged in a more difficult attentional search, this increases activity and strengthens functional connectivity among IPS, FEF, medial, and lateral frontal areas and anterior insular areas ( namely the multiple demand network [67]). Therefore, analyzing attentional processes without a time-locked approach allows us to highlight mainly the component of brain activation related to the cognitive demand [73].

However, real brain functioning is not a static mechanism: neural mechanisms are constrained by connectivity. Therefore, brain connectivity is crucial to clarifying how neurons and neural networks process information. A descriptive analysis of the connectivity patterns showed that ventral attention network, and in particular rTPJ, is strongly connected to dorsal attention networks in the low condition in the theta band. This could be explained by the fact that TPJ plays a more prominent role in attentional shifts when attention and eye movements are dissociated or directed to the periphery of the visual fields [74], a phase that could happen very often in the case of preattention because the subject can easily perceive the target also with the peripherical view. During the high attentional demand level, the main connections are those between IPS and visual areas. The analysis of connection strength confirmed quantitatively that the involvement of visual areas in the high attentional demand condition is significantly higher than in the low condition, probably due to the fact that DAN is expected to exert a greater influence on visual areas in the high condition compared to the low condition and the strength of those connections increases [37,41]. To quantitatively test the hypothesis of the lower segregation of the brain network associated with the high attentional demand, the participation index has been computed because it allows analyzing the role of the node considering also their belonging to a specific subnetwork [75]. The number of connections linking dorsal networks, and in particular rIPS, and visual areas are significantly higher in high conditions compared to low conditions, an indication that IPS exerts an influence on the visual area during spatial orienting [36] and in top-down processes [37]. The effects of top-down mechanisms in high attentional demand are statistically significant, even after the FDR correction for multiple comparisons, whereas we cannot speculate upon those related to the low attentional demand for the lack of significant results after FDR correction. This aspect could be another indication that analyzing attentional processes without a time-locked approach enhances the effects due to task difficulty, allowing the observation, not only of the spatio-spectral distribution typical of high attentional demand, but also its related strengthened connectivity.

To examine whether there is an association between the brain activity and eye blink variations, we analyzed the Spearman’s rank correlation coefficient between spatio-spectral brain features and the EBR of each subject computed binwise. To face the expected type I statistical error, the Descriptive Data Analysis procedure, based on the definition of Ruger’s area for neurophysiological data, was performed [65]. We observed that EBR highly and positively correlates with frontal activity in the theta and beta bands, whereas it negatively correlates with parietal and occipital activity in the alpha band. On the one hand, a decrease in eye blink rate has been previously associated with an increase in activation of the parieto-occipital cortex, which has been demonstrated to play a key role in processing visual stimuli [76,77]. On the other hand, subjects presenting a higher frontal theta also have a higher number of blinks, which could be interpreted as a greater perception of task difficulty [31,34] and consequently a lack of concentration on the task [78]. 

Looking at the intrasubject association, we employed the repeated measures correlation due to high statistical power, estimating the association shared among individuals. We observed a negative and moderate correlation between FEF and rIFG activation and eye blink rate, coherently with the expected effects of the high attentional demand condition on both frontal brain areas and eye activity [31,41]. Moreover, this association has been confirmed also by the association between eyeblink rate and brain network features: the repeated measures correlation showed a significant moderate [79] negative correlation (R = −0.62) between EBR and the participation of rIPS toward the other subnetworks. These findings suggest that top-down mechanisms associated with the high attentional demand condition induce a decrease in the number of blinks, an increase in frontal area activation, and an increase in connectivity between dorsal attention network and visual areas; moreover, such variations are correlated.

Therefore, these results imply that the simple model that can be obtained through the application of just one electrode on the forehead monitoring the eye blinks can give a piece of information that is significantly correlated to the modulation of brain activity and connectivity related to attentional demand variations.

If, on the one hand, the progress done in the neurotechnological field has allowed the development of easy-to-wear and easy-to-use biosignal acquisition systems for this purpose [19], on the other hand, it has given rise to questions about their acceptability [9]. According to Nielsen′s model [80], the acceptability of a system passes through its utility and usability. While the usefulness of attention monitoring is related to the necessity to increase safety during certain activities (e.g., driving a car), its usability depends heavily on factors such as ease and pleasantness of use and reliability [81,82,83]. For this reason, it is necessary to adapt computational models for the analysis of neurophysiological correlates of attention to the practical needs already evidenced, avoiding the use of systems with too high instrumental complexity.

In this regard, the possibility of using other techniques to recognize the ocular activity, instead of EOG, and even less invasive methods, such as eye-tracking or video recording, that could ideally be employed in some operational or daily life activities (e.g., control rooms, car driving), maximizing in that regard not only the utility of this eye activity-based model but also its usability, must be mentioned.

Notwithstanding these encouraging results, this study has a few limitations. First of all, the limited sample size. For this reason, the non-parametric statistic has been preferred over all the performed analyses, even if for more powerful results, the number of subjects needed to be increased. The ocular activity has been described in terms of eye blinks; however, other aspects should be analyzed for a more complete analysis. Moreover, the cortical activity has been reconstructed by means of sLORETA method and it has not been tested and compared against other methods like, for example, eLORETA, which could give different values of reconstructed signals. Finally, the brain networks description could be refined by analyzing other indexes of the graph theory, such as those describing other local and global network features [84,85].

## 5. Conclusions

This work fits into the framework of the joint analysis of eye blinks and brain activities, to provide a comprehensive neuroscientific model to investigate the association between brain mechanisms and manifest behavior during different levels of attentional demand. In this regard, as a manifest aspect of attention-related behavior, eye blinks have provided information correlated to both brain spectral and network features, suggesting the EBR is a reliable measure of attentional demand, especially for out-of-the-lab applications.

## Figures and Tables

**Table 1:** Coordinates of selected ROIs. R and L define right and left brain hemisphere.

ROI	X	Y	Z
LIFG	−36	16	−4
RIFG	42	18	−6
LTPJ	−52	−54	23
RTPJ	51	−54	26
RFEF	20	−13	53
LFEF	−22	−13	55
RIPS	39	−42	51
LIPS	−42	−36	45
RVIS	36	−81	−13
LVIS	−35	−81	−13

**Table 2:** Wilcoxon Signed-Rank Test results for eye blinks parameters.

Parameter	Low Mean(Std)	High Mean(Std)	*Z*	*p*
*EBR*	19.88(10.30)	15.604(9.21)	3.17	0.004
*Duration*	0.221(0.046)	0.217(0.036)	0.86	0.386

## Data Availability

Data available on request due to privacy restrictions.
